# A Review of Techniques Used for Induction Machine Fault Modelling

**DOI:** 10.3390/s21144855

**Published:** 2021-07-16

**Authors:** Carla Terron-Santiago, Javier Martinez-Roman, Ruben Puche-Panadero, Angel Sapena-Bano

**Affiliations:** Institute for Energy Engineering, Universitat Politècnica de València, Camino de Vera s/n, 46022 Valencia, Spain; cartersa@alumni.upv.es (C.T.-S.); jmroman@die.upv.es (J.M.-R.); rupucpa@die.upv.es (R.P.-P.)

**Keywords:** analytical models, fault diagnosis, induction machines, numerical method based models

## Abstract

Over the years, induction machines (IMs) have become key components in industry applications as mechanical power sources (working as motors) as well as electrical power sources (working as generators). Unexpected breakdowns in these components can lead to unscheduled down time and consequently to large economic losses. As breakdown of IMs for failure study is not economically feasible, several IM computer models under faulty conditions have been developed to investigate the characteristics of faulty machines and have allowed reducing the number of destructive tests. This paper provides a review of the available techniques for faulty IMs modelling. These models can be categorised as models based on electrical circuits, on magnetic circuits, models based on numerical methods and the recently proposed in the technical literature hybrid models or models based on finite element method (FEM) analytical techniques. A general description of each type of model is given with its main benefits and drawbacks in terms of accuracy, running times and ability to reproduce a given fault.

## 1. Introduction

**Figure 1:**
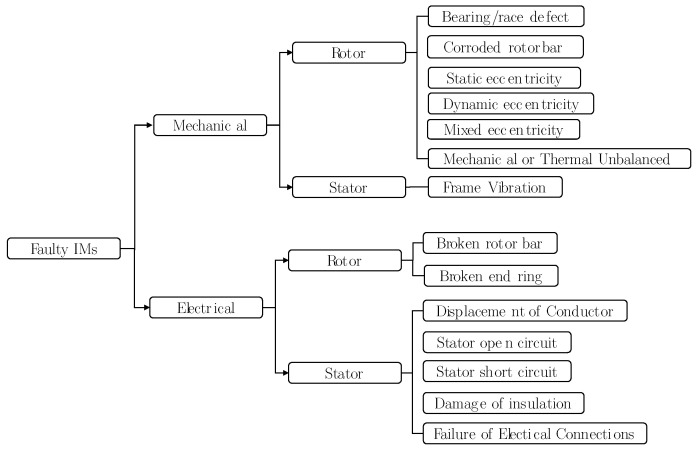
Summary of different types of faults in induction machines [12].

**Figure 2:**
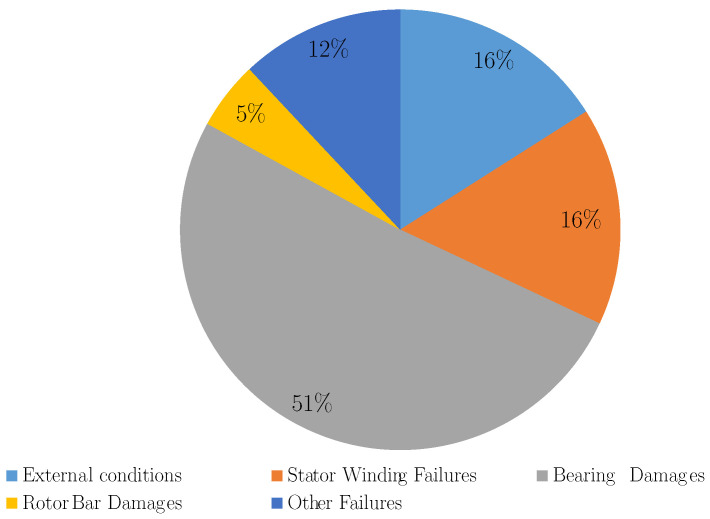
Pie chart for common reasons for downtimes of IMs.

Induction machines (IMs) are widely used in industry applications because of their reduced cost, robustness and reliability. However, induction machines (IMs) are not free from failure. The main sources of faults in IMs can be internal, external or due to environmental conditions. Various perspectives can be found in the literature to categorise the IMs faults. For example, internal faults are usually classified according to their origin, i.e., electrical and mechanical, or to their outbreak location, i.e., stator and rotor [1]. Figure 1 shows a fault tree where faults in IMs are categorised according to their origin (mechanical or electrical) and location (stator or rotor). Some of these failures cause downtimes of the machines which could cause unexpected stoppages leading to important economical loses. Figure 2 illustrates the common reasons for downtimes of IMs. As can be seen, bearing related failures are responsible for 51% of the downtime of IMs. They are followed by those related to stator winding and external conditions damages, which each account for 16% of faults. Rotor-related failures represent 5% and other damages 12% [2,3]. Bearing- and stator-related faults are the most common type of faults, which together account for over 60% of the downtime of electrical machines. Moreover, defective bearings can increase power consumption of IMs whereas efficiency decreases [4]. As such, the literature focuses on the conditioning monitoring systems and the development of fault diagnosis techniques with the aim to detect these faults at an early stage and to track their evolution. Therefore, the maintenance tasks can be scheduled and the outage time imposed by sudden breakdown can be reduced [5]. With this purpose, there are many published condition monitoring techniques based on the analysis of different magnitudes such as thermal [6], chemical, acoustic [7], torque analysis, induced voltage analysis, partial discharge analysis [8], vibration analysis [9], or motor 35 current signature analysis (MCSA) [10,11]. The reliability of condition monitoring systems is based on understanding the behaviour of the machine in healthy and faulty status working under different conditions. The development of the techniques usually involves the analysis of data coming from simulated models and the identification of fault signatures. However, as IMs are key components, it is not only necessary to identify the presence or absence of a given fault, but it must also be quantified. The severity degree of the fault must be indicated in order to plan maintenance tasks. For this purpose, the use of artificial intelligence applied to the fault diagnosis of IMs has been proposed in the recent years.

There is a rising interest in developing condition monitoring systems based on artificial intelligence techniques such as as support vector machine (SVM) [13], artificial neural networks (ANN) [14], Naïve Bayes classifier, Ensemble, k-nearest neighbours (KNN) or decision trees as they can determine not only the presence or absence but also the severity degree of given fault, which improves the reliability. In fact, they have been used to develop condition monitoring systems able to detect different types of faults such as broken rotor bar [15,16], stator short circuit [17,18] or bearing faults [14,16,19], among others.

Contrary to conventional fault diagnosis techniques, condition monitoring systems based on artificial intelligence do not work as an execution of a sequence of commands that finally generate a solution, but they develop a previous training stage to learn the problem and provide a suitable solution [20], that is, determine the machine status. For this training, these expert systems analyse and interpret a failure representative database to evaluate the machine status.

Therefore, these expert systems need to be trained with a large number of cases, with different fault types, severity degrees and combination of faults, with signals in real-time [17]. These requirements imply the need to access many machines, which is only possible at a limited scale with wide industrial cooperation and, anyway, the number of faulty machines is limited. On the other hand, the IMs installed in laboratory test benches have the same problems: the limitation of machines available, the costs associated with a large number of destructive tests and the difficulty to modify the working conditions and to set the different failures. Numerical method-based approaches allow replicating faulty conditions that cannot be tested in the field or test bench laboratories, providing fault data for machine learning algorithms [15,16]. IM faulty models should consider the detailed structure of the machine to obtain simulations results that accurately reproduce the behaviour of the actual IM. These models must allow monitoring the magnitude required to detect the fault and besides, run in real-time.

In summary, the modelling of faulty IMs would be very useful for examining the operational characteristics of faulty machines, minimising destructive testing as well as validating new fault diagnostic techniques or training and testing condition monitoring systems based on artificial intelligence [20]. Thus, high costs associated with machines and destructive testing would be greatly reduced. These savings will be more pronounced in industry and power generation, where the largest machines are found.

**Figure 3:**
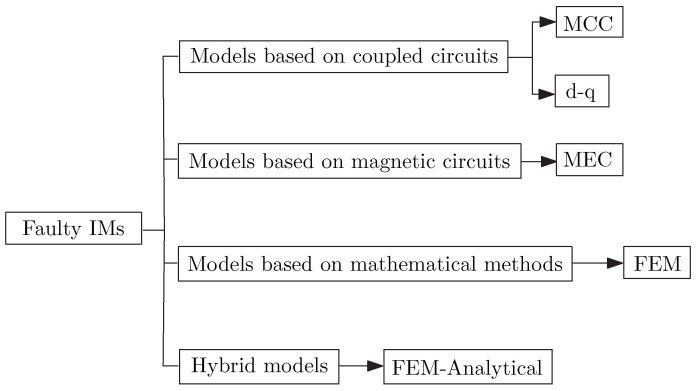
Breakdown of induction motor fault models.

This paper presents a review of the most recent advances in the development of IM faulty models, breaking down into four broad categories, as illustrated in Figure 3. The categories can be further classified as models based on electrical circuits, models based on magnetic circuits, models based on numerical methods and hybrid models. For each type of model, various fault diagnosis methods are covered and a number of summary tables are presented at the end of the subsections dealing with some of the approaches to summarise the references to pertinent works.

The paper is structured as follows. In Section 2, faulty models based on coupled circuits, mainly those based on multiple coupled circuit and d-q transform are reviewed. In Section 3, faulty IM modelling based on magnetic circuits is presented. In Section 4, various models of faulty IMs using numerical techniques and, specifically, those based on finite element method are presented. In Section 5, hybrid numerical-analytical-based techniques for faulty IMs modelling are described. Finally, in Section 6 the main conclusions are presented.

## 2. Models Based on Coupled Circuits

One of the most widely used models based on coupling circuits are those based on the vector space decomposition technique or d-q model. These models assume some assumptions such as fully symmetrical motors, linear iron permeability, air-gap uniformity or the absence of the tangential induction component in the air-gap. All these assumptions allow simplifying the resulting mathematical model. Therefore, it is computed quickly and has enough accuracy for developing control systems. However, in case of the faulty models, these simplifications can be no longer applied as they affect the performance of the faulty machines. The following subsections include a review of the main advances of models based on coupling circuits and on the d-q decomposition.

### 2.1. Multiple Coupled Circuit Models

**Figure 4:**
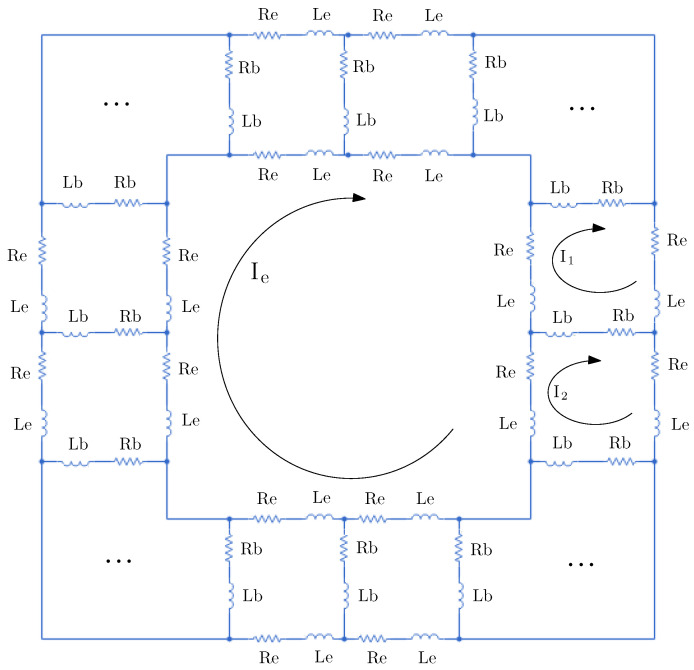
Multiple coupled circuit topology. Equivalent circuit of a rotor cage with multiple-coupled loops for healthy IM.

The detailed modelling procedure as well as the simulation results of models based on coupled circuits are included in [21,22]. The multiple coupled circuit (MCC) models are developed considering that both the stator and rotor are multiple inductive circuits coupled together, with the current in each circuit being an independent variable. Figure 4 shows the rotor cage described as a mesh, where $R_{e}$ and $L_{e}$ are the resistance and leakage inductance of the end ring segment, respectively; $R_{b}$ and $L_{b}$ are the rotor bar resistance and leakage inductance, respectively; $I_{e}$ is the end ring current; and $I_{1}$ and $I_{2}$ are the currents of the first and second rotor loop, respectively. It can be observed that the rotor loop comprises two adjacent rotor bars together with the corresponding end ring segments [23]. Besides, the *n* rotor loop currents are coupled to each other and to the stator windings through mutual inductances. The end-ring loop does not couple with the stator windings; it, however, couples the rotor currents only through the end leakage inductance and the end-ring resistance [24]. Thus, the MCC method can be used to represent a wide variety of fault modes without modification of the model structure shown in Figure 4. In some cases, it only requires the modification of the values of the elements of the circuit in order to introduce the new fault. As an example, a rotor broken bar implies a large value of resistance associated to the broken bar.

However, estimating the parameters of the machine is one of the critical steps in the modelling of faulty IMs. Resistance is usually estimated through the examination of the dimensions of conducting paths. On the contrary, the computation of the coupling elements of a faulty machine is very challenging. There are several methods proposed in the technical literature, with the winding function approach (WFA) being one of the most commonly method used to evaluate the self- and mutual inductances of the stator and rotor circuits [23]. This approach integrates the winding functions to obtain the phase inductances, solving complex integrals in the process, specially in the case of arbitrary winding distributions, which results in a time-consuming task. In an attempt to reduce the computation time, the work in [25] proposes a method based on a single discrete circular convolution, instead of the integrals of the windings functions for every rotor position, in order to obtain the winding inductances. With the proposed approach, the mutual inductances of two phases are calculated for every relative angular position using a single equation which is solved with the fast Fourier transform (FFT). Asymmetrical winding distributions, and the linear rise of the air gap MMF across skewed slots, are easily modelled without increasing the computation time. In fact, the computation of the inductance matrix for the IM given takes just 0.26 s using the proposed method versus more than 7 s with WFA. Therefore, the calculation time is drastically reduced, by a factor approximately 30, while keeping similar accuracy to WFA approach.

On the other hand, although WFA includes the effect of space harmonics, it usually assumes the symmetry of the main magnetic circuit, which makes it unsuitable for the analysis of eccentricities, as shown in [26]. In an attempt to overcome this drawback, the technical literature proposes the modified winding function approach (MWFA). This approach considers air-gap eccentricity for the inductance calculation, allowing to reproduce the effects of static, dynamic or mixed eccentricity in IMs [27]. This approach is not only considered to perform eccentricity faults, authors, such as those in [28], use MWFA to reproduce accurately the air-gap variation according to the bearing fault. In [29], a method based on scaling techniques to compute the parameters for a machine with complex dynamic eccentricity from the inductance curves for healthy, symmetric IM previously computed with the WFA is proposed. These values can then be organised in look-up tables and easily “pulled out” in an iterative procedure of solution of system equations of the model (Equations (5)–(10)). Moreover, in the technical literature, an extension of the MWFA is proposed to include the influence of the rotor skew and the broken rotor bar fault which allows for all harmonics of magneto-motive forces to be taken into account [30].

Once the parameters of the model are known, the expressions (electrical axis attached to the rotor conductors) that define the behaviour of an IM and which have to be solved, for the stator, are

(1) $$\left[U_{s}\right]=\left[R_{s}\right]\left[I_{s}\right]+\frac{d}{dt}\left[\phi_{s}\right]$$

and

(2) $$\left[\phi_{s}\right]=\left[L_{ss}\right]\left[I_{s}\right]+\left[L_{sr}\right]\left[I_{r}\right]$$

where $\left[U_{s}\right]$ is the stator voltage vector, $\left[I_{s}\right]$ is the stator currents vector, $\left[I_{r}\right]$ is the rotor loop current vector, $\left[\phi_{s}\right]$ is the stator flux linkage vector, $\left[R_{s}\right]$ is a diagonal matrix with the stator phase resistances, $\left[L_{ss}\right]$ is the stator windings inductance matrix and $\left[L_{sr}\right]$ is the stator to rotor mutual inductance matrix.

(3) $$\left[L_{ss}\right]\;=\;\left[\begin{array}{ccc} L_{ss11} & L_{ss12} & L_{ss13}\\ L_{ss21} & L_{ss22} & L_{ss23}\\ L_{ss31} & L_{ss32} & L_{ss33} \end{array}\right]$$

where $L_{ssij}$ is the mutual inductance between the stator phase *i* (*i* = 1, 2 or 3) and the stator phase *j* (*j* = 1, 2 or 3). The mutual inductance $L_{sr}$ matrix is an 3 by *n*, where *n* is the total sum of stator phases and rotor loops, matrix comprised of the mutual inductances between stator and the rotor loops:

(4) $$\left[L_{sr}\right]\;=\;\left[\begin{array}{ccccc} L_{sr11} & L_{sr12} & \cdots & L_{sr1n} & L_{sr1e}\\ L_{sr21} & L_{sr22} & \cdots & L_{sr2n} & L_{sr2e}\\ L_{sr31} & L_{sr32} & \cdots & L_{sr3n} & L_{sr3e} \end{array}\right]$$

where $L_{srij}$ is the mutual inductance between the stator phase *i* (*i* = 1, 2 or 3) and the rotor loop *j* (*j* = 1, 2 ... *n*) and $L_{srie}$ the mutual inductance between the stator phase *i* (*i* = 1, 2 or 3) and the end ring.

On the other hand, any rotor loop is mutually coupled with the other rotor loops and with the stator windings. From Figure 4, the voltage equations for the loops can be written as

(5) $$\left[U_{r}\right]=\left[R_{r}\right]\left[I_{r}\right]+\frac{d}{dt}\left[\phi_{r}\right]$$

where:

(6) $$\left[\phi_{r}\right]=\left[L_{rs}\right]\left[I_{s}\right]+\left[L_{rr}\right]\left[I_{r}\right]$$

where $\left[U_{r}\right]$ is the rotor voltages vector, $\left[I_{r}\right]$ is the rotor currents vector, $\left[I_{r}\right]$ is the rotor loops currents vector, $\left[\phi_{r}\right]$ is the rotor flux linkages vector, $\left[R_{r}\right]$ is the rotor resistance matrix and $\left[L_{rr}\right]$ is the rotor inductance matrix. The resistance matrix $\left[R_{r}\right]$ is given by

(7) $$\left[R_{r}\right]\;=\;\left[\begin{array}{ccccccc} 2\left(R_{b}+R_{e}\right) & -R_{b} & 0 & \vdots & 0 & -R_{b} & -R_{e}\\ -R_{b} & 2\left(R_{b}+R_{e}\right) & -R_{b} & \vdots & 0 & 0 & -R_{e}\\ \vdots & \vdots & \vdots & \vdots & \vdots & \vdots & \vdots \\ \vdots & \vdots & \vdots & \vdots & \vdots & \vdots & \vdots \\ \vdots & 0 & 0 & \vdots & 2\left(R_{b}+R_{e}\right) & -R_{b} & -R_{e}\\ -R_{b} & 0 & 0 & \vdots & -R_{b} & 2\left(R_{b}+R_{e}\right) & -R_{e}\\ -R_{e} & -R_{e} & -R_{e} & \vdots & -R_{e} & -R_{e} & nR_{e} \end{array}\right]$$

The inductance between rotor phases matrix can be written by

(8) $$\left[L_{rr}\right]\;=\;\left[\begin{array}{ccccccc} L_{11}+L_{be} & L_{12}-L_{b} & L_{13} & \cdots & L_{1(n-1)} & L_{1n}-L_{b} & -L_{e}\\ L_{21}-L_{b} & L_{22}+L_{be} & L_{23}-L_{b} & \cdots & L_{2(n-1)} & L_{2(n-1)} & -L_{e}\\ \vdots & \vdots & \vdots & \cdots & \vdots & \vdots & \vdots \\ \vdots & \vdots & \vdots & \cdots & \vdots & \vdots & \vdots \\ L_{(n-1)1} & L_{(n-1)2} & L_{(n-1)3} & \cdots & L_{\left(n-1)(n-1\right)}+L_{be} & L_{(n-1)n}-L_{b} & -L_{e}\\ L_{n1}-L_{b} & L_{n2} & L_{n3} & \cdots & L_{n(n-1)}-L_{b} & L_{nn}+L_{be} & -L_{e}\\ -L_{e} & -L_{e} & -L_{e} & \cdots & -L_{e} & -L_{e} & nL_{e} \end{array}\right]$$

where $R_{b}$, $L_{b}$ are the rotor bar resistance and inductance, $R_{e}$, $L_{e}$ are the end ring segment leakage resistance and inductance, $L_{kk}$ is the self inductance of the kth rotor loop, $L_{b}$ is the rotor bar leakage inductance and $L_{ik}$ is the mutual inductance between rotor loops *i* and *k*, and $L_{be}=2\left(L_{b}+Le\right)$.

The electromagnetic torque generated by the machine, $T_{e}$, is given by

(9) $$T_{e}=I_{s}^{\prime }\left(\frac{d}{d\theta_{r}}\left[L_{sr}\right]\right)I_{r}$$

Finally, the mechanical behaviour is modelled by the following equation:

(10) $$T_{e}-T_{L}=J\frac{d^{2}\theta_{r}}{dt^{2}}+B\frac{d\theta_{r}}{dt}$$

where $T_{L}$ is the mechanical load torque, *J* is the inertia moment, B is the friction coefficient and $\theta_{r}$ is the rotor angular position.

The MCC method has been used to developed different types of stator and rotor faults. Moreover, it allows reproducing unrelated faults without the modification of the model structure. Table 1 provides a list of references for commonly reported faults where the procedure to include the given fault in the model based on coupling circuits is detailed. As an example, the authors of [27] develop a general winding machine with this approach, taking into account all space harmonics without any restriction concerning the the symmetry of the stator or rotor windings. Therefore, the model proposed can be applied to analyse a complex dynamic problem such as dynamic eccentricity. Furthermore, the authors [31] focus their attention on MWFA to study the effects of simultaneous presence of static eccentricity and broken rotor bars on the stator current spectrum. Other authors, such as those in [23], propose fault and healthy MCC-based models to reproduce stator and rotor faults. However, this approach considers some assumptions that can affect the accuracy of the results, such as that the air gap is uniform, the machine has no eccentricity, rotor bars are insulated to each other or there are not inter-bar currents.

On the other hand, with the purpose of modelling the progression of the fault, the work in [32] presents a corrosion model of a faulty rotor bar progress. This model considers the changes of the leakage inductance and resistance of the rotor during the progression of the fault, which affect the harmonic components of stator currents. Moreover, the simulations take a reasonably amount of time, about 30 min to perform.

In summary, MCC modelling and their variants, such as WFA/MWFA, take into account the geometry and winding layout of the machine without any restriction concerning either the symmetry of the stator windings or rotor bars. Moreover, the effect of space harmonics is considered. For these reasons, these models are specially suitable for the analysis of IMs with arbitrarily connected windings and unbalanced operating conditions [33]. On the other hand, although it is usual to disregard some phenomena such as saturation, skin effect, proximity effect and capacitance between windings, due to their complexity, there are some variants that also consider some of these phenomena [34]. Regarding time requirements, especially when compared to numerical-based methods, the work in [25] reports differences of 3 h using finite element method (FEM) versus 7.6 s for the same analysis using WFA. Furthermore, the work in [35] compares the results for eccentric cage IM using FEM and MWFA, showing differences of 50 h versus 4 min for 1.5 s of actual machine run time. Thus, although these analytical models are not as accurate as numerical based models, their lower calculation time becomes them remarkable for fault diagnosis purposes, especially in hardware in the loop (HIL) systems [36]. The greater accuracy that these models provide is achieved at the cost of greater model complexity and higher requirements in both time and computing power [37], limiting its application for on-line fault diagnosis systems and condition monitoring systems based on artificial intelligence (AI).

### 2.2. d-q Models

One of the most commonly used modelling approaches for IMs is d-q modelling, which arose with the aim of simplifying MCC models. These models were developed using orthogonal components of voltages and currents by the Clark and Park transforms. Thus, the expressions of the voltage equations of the IM as well as the torque equation can be transformed from the abc frame to the reference frame dq, where the machine equations are therefore expressed in complex d-q variables [47]. Traditionally, the technical literature has proposed parameter estimation techniques to identify the main parameters of d-q models based on the analysis of data coming from the machine [48,49]. They analyse data such as voltages or currents, under specific working conditions: steady-state or start-up transients [50,51]. These models are commonly used for control drive purposes, which requires testing to obtain signals of the machine. Nevertheless, this procedure could not be the most suitable for fault diagnosis purposes as for each faulty model a faulty machine test is required [50,52]. This means that it requires a large number of destructive tests with its associated costs to obtain the required wide variety of faulty models for the development of fault diagnosis techniques and condition monitoring systems. Therefore, with the aim of reproducing faults with this kind of models, the same parameter estimation techniques are typically used as in the case of the MCC models. The main advantage of this type of models is that the number of equations required for simulation is reduced, as the use of the space vector transformation allows to represent any induction machine with structural symmetry using only four coupled differential equations [53].

Thus, the stator voltage equations are defined by

(11) $$\left[v_{ds}\right]=\frac{1}{w_{base}}\frac{d\phi_{ds}}{dt}-\omega \phi_{qs}+R_{s}i_{ds^{\prime }}$$

(12) $$\left[v_{qs}\right]=\frac{1}{w_{base}}\frac{d\phi_{qs}}{dt}-\omega \phi_{qs}+R_{s}i_{qs^{\prime }}$$

(13) $$\left[v_{0s}\right]=\frac{1}{w_{base}}\frac{d\phi_{0s}}{dt}+R_{s}i_{0s^{\prime }}$$

where $w_{base}$ is the per-unit base electrical speed; $\phi_{ds}$, $\phi_{qs}$ and $\phi_{0s}$, are the d-axis, q-axis and zero-sequence stator flux linkages, respectively; $R_{s}$ is the stator resistance; and $i_{ds}$, $i_{qs}$, and $i_{0s}$ are the d-axis, q-axis, and zero-sequence stator currents, respectively.

The rotor voltage equations are obtained from the expressions:

(14) $$\left[v_{dr}\right]=\frac{1}{w_{base}}\frac{d\phi_{dr}}{dt}-\left(\omega -\omega_{r}\right)\phi_{qr}+R_{rd}i_{dr}$$

(15) $$\left[v_{qr}\right]=\frac{1}{w_{base}}\frac{d\phi_{qr}}{dt}-\left(\omega -\omega_{r}\right)\phi_{dr}+R_{rd}i_{qr}$$

where $v_{dr}$ and $v_{qr}$ are the d-axis and q-axis rotor voltages, $\phi_{dr}$ and $\phi_{qr}$ are the d-axis and q-axis rotor flux linkages, ω is the per-unit synchronous speed, $\omega_{r}$ is the per-unit mechanical rotational speed, $R_{rd}$ is the rotor resistance referred to the stator, and $i_{dr}$ and $i_{qr}$ are the d-axis and q-axis rotor currents. The rotor torque, *T*, is defined by

(16) $$\left[T\right]=\left[\phi_{ds}\right]\left[i_{qs}\right]-\left[\phi_{qs}\right]\left[i_{ds}\right]$$

A more detailed description can be found in [52]. Therefore, these models can be run very fast and, furthermore, they can be easily implemented in real-time hardware simulator systems (HIL) [54]. Regarding the development of faulty IM models, d-q models are widely used to simulate transient and steady-state phenomena, as well as to reproduce phase unbalances or oscillatory torque during start-up [55]. However, each fault case requires a modification in the model structure [33]. Besides, the faults considered are external and not from the machine itself, and, thus, they are almost unsuitable for fault diagnosis purposes. With the aim of including internal faults, the authors of [56] propose the use of the multiple reference frames theory for the diagnosis of stator faults. This approach allows the extraction and manipulation of the information contained in the motor supply in a way that the effects of faults can be measured and isolated.

Typically d-q models are used to study rotor bar faults. Nevertheless, the technical literature proposes this approach to study stator shorts circuits as well as eccentricity and bearing faults. In Table 2 can be found a reference list for the various types of faults studied using d-q modelling. As an example, the authors of [57] propose the development of a comprehensive set of d-q based algorithms with fault simulation and fault diagnosis purposes. This approach is used to study eccentricities and to compare a single broken bar with other breakages such as broken bars and broken connectors, reporting simulation times of 36 min for an average of 4 s. However, although d-q modelling reduces the number of equations required for simulation, it does not use nor provide any information about individual rotor bars or end rings currents.

In general terms, these models assume both uniformity in the air gap and that the electromotive force is sinusoidally distributed along the air gap. Besides, they do not include the effect of spatial harmonics making these models poorly suited to be used in diagnostic algorithms. The time and space harmonics have impact on speed, torque, currents and other performance parameters of electrical machines, whereby it is a very limited option for developing on-line condition monitoring systems.

## 3. Models Based on Magnetic Circuits

Contrary to MCC approach, based on coupled electrical circuits, magnetic equivalent circuit (MEC) models are based on detailed magnetic modelling obtaining the machine’s model by approximations of a network of reluctances and permanences.

**Figure 5:**
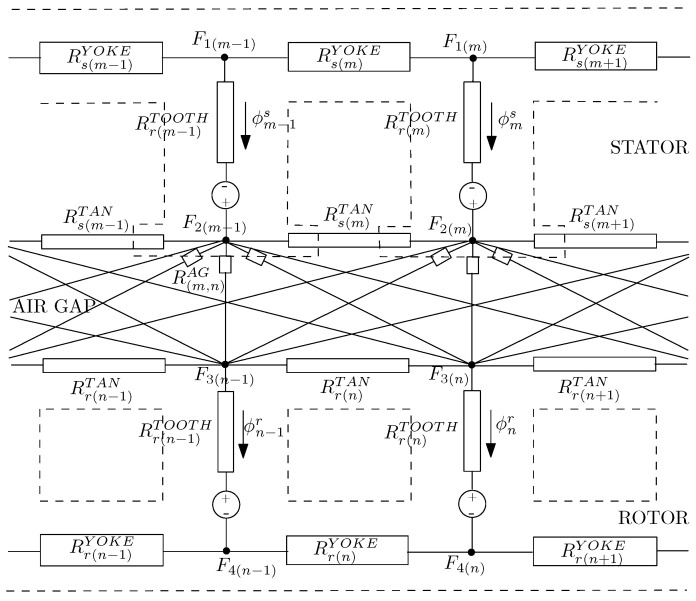
Simplified magnetic equivalent topology for healthy IM with closed rotor slot [67].

Figure 5 illustrates a simplified MEC model of the induction machine. The MEC model is assembled such that every tooth on the stator is coupled to every tooth on the rotor, and vice versa. Air-gap reluctances depend on the relative position of the corresponding stator and rotor teeth [67]. The magnetic equivalent circuit includes closed loop paths containing rotor and stator teeth fluxes, $\phi^{r}$ and $\phi^{s}$, respectively. Furthermore, $R_{r}$ and $R_{s}$ are the stator and rotor teeth reluctances, respectively. The index, *m*, identifies a stator tooth and the index, *n*, identifies a rotor tooth. $F_{1}$, $F_{2}$, $F_{3}$ and $F_{4}$ correspond to the magneto-motive nodal forces, and $F_{s}$, $F_{r}$ correspond to the stator and rotor tooth magneto-motive forces. Contrary to MCC, where resistances and inductances must be estimated, in the case of MEC models, reluctances, *R*, or permeances must be obtained, usually via geometry calculations. The magneto-motive forces can be approximated through FEM-based simulations or the phase currents [68]. These permeances are expressed as functions of the machine geometry and the instantaneous fluxes. Thus, this approach allows to incorporate space-harmonics associated to discrete winding distributions, stator and rotor slotting and saliency effects caused by saturation of the magnetic materials [33]. The rotor and stator fluxes [ϕ] are related to the nodal magneto-motive forces [F] by the reluctances [R], from the equation

(17) [ϕ]=[R][F]

On the other hand, the stator and rotor tooth magneto-motive forces are computed through the following expressions:

(18) $$\left[F_{s}\right]=\left[F_{2}\right]-\left[F_{1}\right]+\left[R_{s}\right]\left[\phi^{s}\right]$$

(19) $$\left[F_{r}\right]=\left[F_{3}\right]-{\left[F_{4}\right]}^{T}-\left[R_{r}\right]\left[\phi^{r}\right]$$

The expression for the electromagnetic torque, $T_{em}$, can be derived from the equation

(20) $$\left[T_{em}\right]=\frac{1}{2}\sum_{m=1}^{N_{s}}\sum_{n=1}^{N_{r}}{\left(F_{2_{m}}-F_{3_{n}}\right)}^{2}\frac{dP_{AG_{\left(m,n\right)}}}{d\theta_{r}}$$

where, $\theta_{r}$ is the rotor position in rads and $P_{AG_{\left(m,n\right)}}$ is the air-gap permeance between the *m* stator tooth and the *n* rotor tooth.

The main difficulty of this type of modelling is to include the air gap permeance between a stator and a rotor tooth, sd it is influenced by fringing. To overcome this, some authors propose to divide the permeance in four parts: a non-interaction part, two partially overlapping parts and a constant part [69]. On the other hand, other authors, such as those in [70], just exclude the fringing effect in the air gap permeance to reduce computation complexity obtaining a model able to run in a real-time simulation while keeping reasonable accuracy. Usually, due to the models characteristics, they require long simulation times. However, in [71], a MEC model suitable for real-time simulation of IMs is proposed. To do this, the permeances are defined as a nonlinear function of magnetic scalar potentials due to iron saturation effect. Thus, the resulting model reduces the traditionally long simulation times and can be employed in the HIL test setup.

Regarding faulty IM’s modelling, various IM faults have been modelled by the MEC approach, as shown in Table 3. However, very few works have been reported in the recent years for the case of bearings and eccentricity faults. As an example, the work in [72] uses the MEC for the study of defective rolling bearings. For this purpose, the MEC model of the IM is developed by dividing the uniform distribution parts into a certain number of flux tubes. A magnetic equivalent network is formed by connecting these flux tubes with nodes. The defects in outer/inner raceways and rolling balls are, respectively, simulated by half-wave sinusoidal functions.

In the case of eccentricity, the authors of [3] propose the implementation of a modified expression for the air gap permeance and includes the effects related to eccentric rotor positions in the development of their MEC model. These air-gap permeances represent a continuously changing permeance as a function of the rotor position. Alternatively, the authors of [73] develop a MEC model for real-time study of various faulted IMs. A discretisation method in time-domain is utilised to keep the MEC coefficient matrix unchanged during nonlinear iterations, in order to overcome the timing constraints of real-time simulation due to the nonlinearity and rotation of electric machines.

According to the literature, the MEC approach has the advantage of moderate computational complexity, especially when it is compared to the high accuracy modelling, but the accuracy during transient conditions is limited because it does not usually include distributed circuit effects in the rotor conductor or the stator ring leakage inductance [74,75]. For example, in [74] the computing complexity is reduced by a single set of equations which includes magnetic and electric equations. Thus, this model can simulate the healthy and faulty machine under various kinds of faults by a single model, reducing the complexity of equations and the simulation time of the the conventional MEC approach. Similarly, the work in [76] compares the the processing time for the same analysis for the proposed method and conventional MEC. This work reports processing times of more than 100 min for conventional MEC approach versus 64 min for the proposed method, which means a computation time improvement of about 39%. Furthermore, if compared to FEM based, the resulting computation time is much shorter, about 5%. More recently, the work in [75] reports differences of 70 h for a FEM analysis versus 18 min for the same analysis carried out via MEC approach. The MEC model achieves savings in computational costs of more than 97% when compared with the FEM-based model while keeping good accuracy. To conclude, the MEC-based approach can be reasonably accurate in predicting machine performances over a range of operating points and load conditions as well as unbalanced excitation and faulty conditions, being considered a good compromise between the standard lumped parameter models and FEM based in terms of computation time and accuracy [70].

## 4. Models Based on FEM

Circuit-based models run fast but cannot provide comprehensive modelling as the field models. Techniques that can take into account the nonlinearities of the magnetic materials, as well as to avoid simplified assumptions about the geometry and arrangement of the windings, have been proposed. Numerical techniques based on FEM or boundary elements method, BEM, consider the above and can be used to accurately reproduce the performance of the induction machine [79]. Among them, FEM is the numerical method most reported in the technical literature, which can serve as a feasible approach in fault diagnosis of IMs.

This method uses the exact magnetic and geometric characteristics of the machine to compute their magnetic field distribution. This magnetic field distribution within the IM contains accurate information on the stator, rotor and the mechanical parts of the machine [80]. Moreover, it allows to calculate the machine parameters such as the magnetic flux density, inductances and electromagnetic torque including spacial harmonic effects and split winding pattern. Therefore, mostly IM faults can be reproduced by the monitoring the magnetic fields [81,82]. Faulty IM models are usually developed in 2D, which has the advantage of being very accurate in terms of magnetic phenomena. However, these models do not include the skewing effect of the rotor, the end rings are disregarded and interconnection of the rotor bars is usually accounted for through the electrical circuit, considering the current source ideal [83].

The magneto-dynamic field problem for a general IM in 2D is expressed by the expression [84,85]

(21) $$\frac{d}{dx}\left(\frac{1}{\nu }\frac{dA_{z}}{dx}\right)+\frac{d}{dy}\left(\frac{1}{\nu }\frac{dA_{z}}{dy}\right)=-J_{0}+\sigma \frac{dA_{z}}{dt}-\sigma \vec{v}x\left(\nabla x\vec{A}\right)$$

where $\vec{A}$ is the magnetic vector potential, $A_{z}$ is the z-component of the magnetic vector potential, $J_{0}$ is the applied density current source, $\vec{v}$ is the velocity, σ is the electric conductivity and ν the permeability.

The magnetic flux density field, $\vec{B}$, is obtained from the expression

(22) $$\vec{B}=\nabla x\vec{A}$$

Subsequently, the forces are computed via the Maxwell stress tensor. It can be applied of in the modelling of IMs with fault and unbalanced cases. In recent years, the technical literature has provided a wide variety of faulty IM models based on FEM. Table 4 gives references for some typical faults. As an example, in [86] a fluxgate sensor is used to detect broken rotor bar fault pattern via the radial leakage flux using a 2D-time stepping finite element method (2D-TSFEM).

The proposed method and the traditionally used motor MCSA are compared to each other, stating that the proposed method is more accurate than the classical motor stator current analysis of the IMs [87]. However, the fluxgate sensor is challenging to use at the practical level. On the other hand, recently in [88] the effects of static eccentricity in electromagnetic parameters such as voltage, speed, torque, flux density and flux distribution for a faulty motor are accurately represented through TSFEM.

Although these models often produce better results in terms of accuracy, they require a significant computational capacity. Differences of 8 h for a FEM analysis versus 1 min for the same analysis using WFA have been reported in [89]. Even with modern processors, the computational effort required to complete FEM evaluation is notable [90]. Besides, they take long simulation times (from days to even weeks depending on the type of fault) for short time simulated periods [79]. On the other hand, the use of the machine symmetry, which would reduce meshing and computing time, cannot be applied in the case of faulty models. These constraints are even worse when a 3D analysis is performed, where the mesh increases in several orders of magnitude and therefore, simulation times increase exponentially [82]. These drawbacks of the use of the FEM approach are limited in some fault diagnosis fields, such as the development of on-line CM systems or AI-based fault diagnosis systems, which require a wide range of scenarios for different degrees of fault and combination of several types of fault. The evaluation of each scenario requires the full simulation of the new FEM model, with their corresponding long simulation times and high computational costs. Running these models in hardware simulators, which would allow reducing simulation times, is still challenging.

## 5. Hybrid Models

As mentioned above, the modelling based on FEM is very accurate but requires much computing power and long running times, especially as compared with analytical models. However, analytical models assume some simplifications which affect their accuracy often render them unsuitable for fault diagnosis purposes. In an attempt to overcome this, the technical literature proposes the combination of FEM-analytical approaches to obtain models which can be run in real-time simulators with FEM level accuracy [106].

These models use FEM to preset the analytical model parameters accurately, allowing them to be run in real-time simulators, which is a need for fault diagnosis purposes [107,108]. For example, in [82] a hybrid model based on d-q approach, through Equations (11)–(16), and finite element analysis is developed for looking into short circuit faults in IM drives. It proposes the integration of the model with real-time simulators. On the other hand, in [109] an analytical model with the accuracy of FEM is proposed. The sparse identification technique is used to reduce the number of FEM simulations required for the computing of the IM coupling parameters. The coupling parameters obtained are the ones used in the analytical model developed from MCC expressions (5)–(10), which is implemented in a real-time simulator for testing different severity degrees of static eccentricity. Thereby, simulation times and memory resources are significantly reduced. Similarly, the authors of [110] solve through FEM the complete geometry of the IM to compute the coupling parameters and then, importing these parameters in the analytical model of the machine. In this case, FEM analysis run on multiple processor cores working in parallel with each other in order to speed up the simulations. Despite the improvements, these approaches still requires a large number of simulations and memory resources to obtain the coupling parameters.

In an attempt to overcome these issues, the authors of [111] propose the sparse identification to obtain a faulty IM model, reducing the FEM simulations required while keeping good accuracy. Savings, in terms of computational capacity, from more than 13 GB using FEM analysis to 5 MB with the proposed method are reported. This represents a reduction in computational costs of more than 99.9%. However, it still require the full FEM analysis for every fault scenario, with their corresponding long simulation times and high computational costs. Differences of more than 10 h for a FEM analysis versus 25 min for the same analysis carried out via a method based on computational mathematics is reported in [112]. This method allows to avoid the need of a FEM simulation for every new sampling point in the case of static eccentricity fault, reporting time savings of 95.83% when compared to fully FEM simulations. These savings could be larger still in the case of TSFEM-based models compared to the hybrid models. As mentioned in the previous section, TSFEM-based models take long simulation times for short simulated spans, from days to even weeks depending on the type of fault. By contrast, the development of hybrid models to be run in real time platforms and implemented using computational reduction techniques can take about 25 min. Even adding the time to run one simulation (the same as the simulation time span being real time models) the time savings are over 98%.

## 6. Conclusions

Accurate representation of faulty IMs is crucial for research and development in the area of condition monitoring to reduce the limitations of test benches. In this article, four research strategies for IM fault modelling in the literature are reviewed: models based on electrical circuits, models based on magnetic circuits, models based on numerical methods and hybrid models. Nonlinearities and non-idealities of the IMs cannot be properly modelled using circuit-based models. On the other hand, although models based on numerical methods are more comprehensive, they require a significant computational capacity and long simulation times. Factors such as the size or the information available of the machine can influence in the modelling technique applied. Nevertheless, for fault diagnosis purposes, accuracy is one of the factors that can affect the most. There is a real need to establish a model which offer a good balance between accuracy and computation time. Thus, both models based on circuits and models based on numerical methods have limitations that the technical literature tries to overcome with the hybrid approach, obtaining promising results. Although the hybrid approach is more advanced, still few contributions have been reported in the technical literature so far. The combination of techniques for modelling faulty IMs can help in the development of methods, techniques and diagnosis systems with a substantial cost reduction when compared to the use of physical test benches. However, a cost comparison between both alternatives will lack significance due to two main facts: first, the cost associated with physical test benches largely depends on the rated power of the IM and auxiliary equipment, while for virtual ones it depends mainly on the required model complexity, and second, in real hardware the testing is often limited to a set of fault types and severity degrees and destructive testing results in additional equipment replacement costs, whereas virtual test benches are more flexible and can yield new simulations results at small additional cost.

## Figures and Tables

**Table 1:** MCC reference for different types of faults.

Fault	References
Broken rotor bar	[23,24,30,31,32,38,39]
Broken end ring	[21,40]
Stator open circuit	[41]
Stator short circuit	[23,24,41]
Static eccentricity	[27,35,42]
Dynamic eccentricity	[27,29,42]
Mixed eccentricity	[27,43]
Corroded rotor bar	[32]
Bearing/race defect	[28,44,45,46]

**Table 2:** d-q reference for different types of faults.

Fault	References
Broken rotor bar	[53,58,59,60,61]
Broken end ring	[57,62]
Stator open circuit	[63]
Stator short circuit	[52,53,56]
Static eccentricity	[59,64]
Dynamic eccentricity	[57,59]
Mixed eccentricity	[65]
Bearing/race defect	[66]

**Table 3:** MEC references for common faults.

Fault	References
Broken rotor bar	[67,73,74]
Stator short circuit	[68,74,76,77]
Static eccentricity	[75,78]
Dynamic eccentricity	[75]
Mixed eccentricity	[3]
Bearing/race defect	[72]

**Table 4:** FEM references for different types of faults.

Fault	References
Broken rotor bar	[81,82,83,86,87,91,92,93,94]
Broken end ring	[57]
Stator short circuit	[82,95,96,97]
Static eccentricity	[81,82,88,98,99]
Dynamic eccentricity	[81,100]
Mixed eccentricity	[80,101,102]
Bearing/race defect	[103,104,105]
